# PPAR**γ** Regulates Genes Involved in Triacylglycerol Synthesis and Secretion in Mammary Gland Epithelial Cells of Dairy Goats

**DOI:** 10.1155/2013/310948

**Published:** 2013-04-17

**Authors:** Hengbo Shi, Jun Luo, Jiangjiang Zhu, Jun Li, Yuting Sun, Xianzi Lin, Liping Zhang, Dawei Yao, Huaiping Shi

**Affiliations:** Shaanxi Key Laboratory of Molecular Biology for Agriculture, College of Animal Science and Technology, Northwest A&F University, Yangling, Shaanxi 712100, China

## Abstract

To explore the function of PPAR**γ** in the goat mammary gland, we cloned the whole cDNA of the PPAR**γ** gene. Homology alignments revealed that the goat PPAR**γ** gene is conserved among goat, bovine, mouse, and human. Luciferase assays revealed that rosiglitazone enhanced the activity of the PPAR**γ** response element (PPRE) in goat mammary epithelial cells (GMECs). After rosiglitazone (ROSI) treatment of GMECs, there was a significant (*P* < 0.05) increase in the expression of genes related to triacylglycerol synthesis and secretion: *LPL, FASN, ACACA, PLIN3, FABP3, PLIN2, PNPLA2, NR1H3, SREBF1*, and *SCD*. The decreases in expression observed after knockdown of PPAR**γ** relative to the control group (Ad-NC) averaged 65%, 52%, 67%, 55%, 65%, 58%, 85%, 43%, 50%, and 24% for *SCD, DGAT1, AGPAT6, SREBF1, ACACA, FASN, FABP3, SCAP, ATGL,* and *PLIN3*, respectively. These results provide direct evidence that PPAR**γ** plays a crucial role in regulating the triacylglycerol synthesis and secretion in goat mammary cells and underscore the functional importance of PPAR**γ** in mammary gland tissue during lactation.

## 1. Introduction 

Lactation is a process highly demanding of lipid synthesis and transport. Although peroxisome proliferator-activated receptor *γ* (PPAR*γ*) is known to promote lipogenesis and adipogenesis in adipose tissue [1], its role in the lactating mammary gland is less clear. Many candidate genes that regulate lipid synthesis have been identified during the lactation cycle [2]. Researchers have evaluated the expression profiles of 54 genes associated with bovine milk fat synthesis through various periods during lactation and built a regulatory network [3]. Their data showed that PPAR*γ* might be the main factor that regulates the nuclear transcription factor, sterol regulatory element-binding transcription factor 1 (SREBF1), which also affects the expression of some fatty acid metabolism genes during lactation [3, 4].

Much data have been published regarding PPAR*γ*'s role in milk fat synthesis in bovine [5–7], while there is a lack of data on its role in the dairy goat. Whether PPAR*γ* also plays the same critical role in regulation of milk fatty acid synthesis during the lactation process in dairy goat remains to be determined. In the present study, we first identified the sequence of PPAR*γ* in dairy goat mammary tissue and evaluated the activity of the PPRE via luciferase assays. Its function in dairy goat mammary epithelial cells (GMECs) was also investigated through the use of the pharmaceutical ligand rosiglitazone (ROSI) and adenovirus-mediated RNA interference.

## 2. Materials and Methods

### 2.1. cDNA Cloning

The primers used in the amplification of the goat PPAR*γ* transcript sequence (*PPARG*) used for cDNA cloning are reported in Table 1. Primers were designed based on the consensus conserved sequences between humans (AB472042) and bovines (BC116098). The PCR reaction was performed with goat mammary epithelial cell cDNA as a template. The cDNA cloning of the 5′ and 3′UTR was implemented according to the manufacturer's protocols of the 5′RACE system Ver.2.0 (Invitrogen, USA) and 3′-full RACE core set Ver.2.0 (Takara, Japan). The nested gene-specific primers for *PPARG*, designed based on its open read fragment (ORF), were used for 3′RACE. Similarly, the nested gene-specific primers (Table 1) were also designed for 5′RACE. All the PCR fragments were cloned into pMD-19T plasmid vectors (Takara, Japan) and then sequenced at a commercial facility (Invitrogen, Shanghai, China). The PPAR*γ* protein structure was predicted using PHYRE2 (http://www.sbg.bio.ic.ac.uk/phyre2/html/page.cgi?id=index).

### 2.2. Vector Construction and shRNA

The luciferase vector (pGL3-basic) containing three copies of PPRE was designed as described before [8]. The shRNA sequences were designed using the WI siRNA Selection Program (http://sirna.wi.mit.edu/home.php) and BLOCK-iT RNAi Designer (http://rnaidesigner.invitrogen.com/rnaiexpress/) using the goat PPAR*γ* gene sequence (HQ589347.1). We selected the highest-ranked shRNA sequences. Additionally, a BLAST search against all EST sequences in GenBank was performed to ensure that the selected sequences were specific for goat PPAR*γ*. Meanwhile, those sequences were selected and synthetized at a commercial facility (Invitrogen, Shanghai, China) with *Bam*H I and *Xho* I restriction sites suitable for the cloning process (see Table 2). Lastly, three shRNA were generated by heat treatment annealing and constructed into *pENTR/CMV-GFP/U6*-shRNA. The CDS of PPAR*γ* was subcloned into the *pDsRed1-C1 *plasmid vector between the *Xho* I and *Eco*R I restriction sites to generate *pDsRed1-C1-PPAR*γ**.

### 2.3. Cell Culture and Treatments

Goat mammary epithelial cells isolated from a Xinong Saanen goat at peak lactation [9] were allowed to grow in 60 mm culture dishes (NUNC, Denmark) in DMEM/F12 medium (HyClone, China). Routine cultures were incubated at 37°C in 5% CO_2_ and air. Culture medium was changed every 24 h. Medium was composed of DMEM/F12 with insulin (5 mg/L, Sigma, USA), hydrocortisone (5 mg/L, Sigma, USA), penicillin/streptomycin (10 kU/L, Harbin Pharmaceutical Group, China), epidermal growth factor (1 mg/L, Sigma, USA), and fetal bovine serum (10%, Gibco, USA). ROSI (BioVision, USA) was resuspended in DMSO (Sigma, USA) at a concentration of 50 mmol/L. Cells cultured in 60 mm culture dishes and subcultured to 90% confluence were treated with 50 *μ*mol/L ROSI and harvested at 0, 12, and 24 h after treatment to extract total RNA. The 293A cells for preliminary testing of shRNA and generating recombinant adenovirus were cultured in the basal medium containing 10% fetal bovine serum and 90% DMEM (Gibco, USA).

### 2.4. Preliminary Screening of shRNA Sequences

In order to get the most effective shRNAs for targeting PPAR*γ* gene, an experiment was done as follows. 293A cells at 80% confluence in 12 plates were transiently transfected with 1.0 *μ*g of three *pENTR/CMV-GFP/U6*-shRNAs with *pDsRed1-C1*-PPAR*γ*, at a ratio of 3 : 2 using FuGENE HD Transfection Reagent (Roche, Switzerland). The *pDsRed1-C1*-*PPAR*γ** vector also was transfected alone as a control in the same amount as above. All the steps were performed in accordance with the manufacturer's protocol. The GFP fluorescence was monitored by using a Leica fluorescent microscope (DMI4000B, Germany).

### 2.5. Adenovirus Generation

shRNA expression cassettes with an EGFP reporter gene in the pENTR vector were switched into an adenoviral vector (pAd/PL-DEST) using the Gateway technique (Invitrogen, USA) to generate pAd-shRNA vectors. *Pac* I linearized adenoviral plasmids were transfected into 293A cells to generate the adenovirus. About 8 to 10 days after transfection, the recombinant virus was collected and subjected to two rounds of amplification in 293A cells. The viral titers were determined in transduced 293A cells through GFP expression as previously described [10–12].

### 2.6. Luciferase Assays

To assess the degree of PPAR*γ* activation, goat mammary epithelial cells at 80% confluence in 96-well plates were transiently transfected with 0.08 *μ*g of PPRE×3-Luc reporter plasmid along with a *Renilla* vector (pRL-TK) as a control using the FuGENE HD transfection reagent at a ratio of 25 : 1. After a 24 h recovery period in medium, cells were treated with 0, 10, 25, 50, and 100 *μ*mol/L ROSI. Forty-eight hours later, cells were harvested and lysates were made using reporter lysis buffer (Promega, USA) according to the manufacturer's instructions. Luciferase activity in the cell extract was determined using luciferase assay buffer and luciferase assay substrate according to the manufacturer's protocol (Promega, USA) in a luminometer (BHP9504, China).

### 2.7. Adenovirus Transduction

Goat mammary epithelial cells at 70–80% confluence were transduced with adenovirus supernatant at a multiplicity of infection (MOI) of 200. The medium was replaced with fresh medium 6 h later. The shRNA negative control adenovirus (Ad-NC) was used as a control. Cells were harvested 48 h after transduction.

### 2.8. RNA Extraction and Real-Time RT-PCR (qPCR)

Total RNA was extracted from cells using RNAprep pure cell kit (Tiangen, China). The first-strand cDNA of different treatments was synthesized from 0.5 *μ*g of purified total RNA using the PrimeScript RT kit (Takara, Japan) according to the manufacturer's instructions. Sufficient cDNA was prepared to run all the selected genes (Table 3). Primers were designed to span exon-exon boundaries according to BLAST against bovine genome in order to avoid amplification of genomic DNA using Primer 5.0 software. The specificity of the primers was tested using the same protocol as for qPCR in a simple thermocycler (S1000, Bio-rad, USA), and the PCR product was run in a 15 g/L agarose gel. In addition, a dissociation protocol was performed in the RT-qPCR. Only primers with a single band on the agarose gel, a unique peak in the dissociation curve after the RT-qPCR, and devoid of primer-dimers were selected. The efficiency of each primer pair was tested using a standard curve as previously described [3]. All the amplicons were sequenced in order to assess the right amplified genes. Characteristics of all primers used in the RT-qPCR reaction are described in Table 3. RT-qPCR reactions were performed according to the manufacturer's instructions (SYBR Premix Ex Taq II, Perfect Real Time, Takara, Japan). Glyceraldehyde-3-phosphate dehydrogenase (*GAPDH*) was selected as an internal control gene [13]. Although we did not verify additional genes as internal controls, *GAPDH* was used partly because it has been used previously in a goat mammary tissue study [13], and also because it has been widely used as the sole control gene in bovine cell studies [14]. However, we understand the limitation of using a single internal control gene because more reliable data requires the verification and use of at least 3 internal controls [15].

### 2.9. Western Blot

Whole cell proteins were extracted with RIPA buffer (Solarbio, China) supplemented with PMSF (Pierce, USA). Western blotting was performed using the following primary and secondary antibodies: anti-PPAR*γ* (Abcam, ab19481, Hong Kong, 1 : 400) and goat anti-rabbit IgG (Tiangen, China, 1 : 1000). All antibodies were used according to the manufacturer's recommendations. Signals were detected using the chemiluminescent ECL Western blot detection system (Pierce, USA).

### 2.10. Statistical Analysis

Each treatment was replicated 3 times, and results are expressed as mean ± SD. Data of RT-qPCR was analyzed relative to the control using the 2^−ΔΔCt^ method. The statistical significance for ROSI treatment was determined by the ANOVA test using SPSS 19.0 software. Treatment means for shRNA interference were separated using Fisher's least significant difference pair-wise comparisons. Significance was declared at *P* < 0.05.

## 3. Results and Discussion

### 3.1. Molecular Cloning and Sequence Analysis of Dairy Goat PPAR*γ*

**Figure 1:**
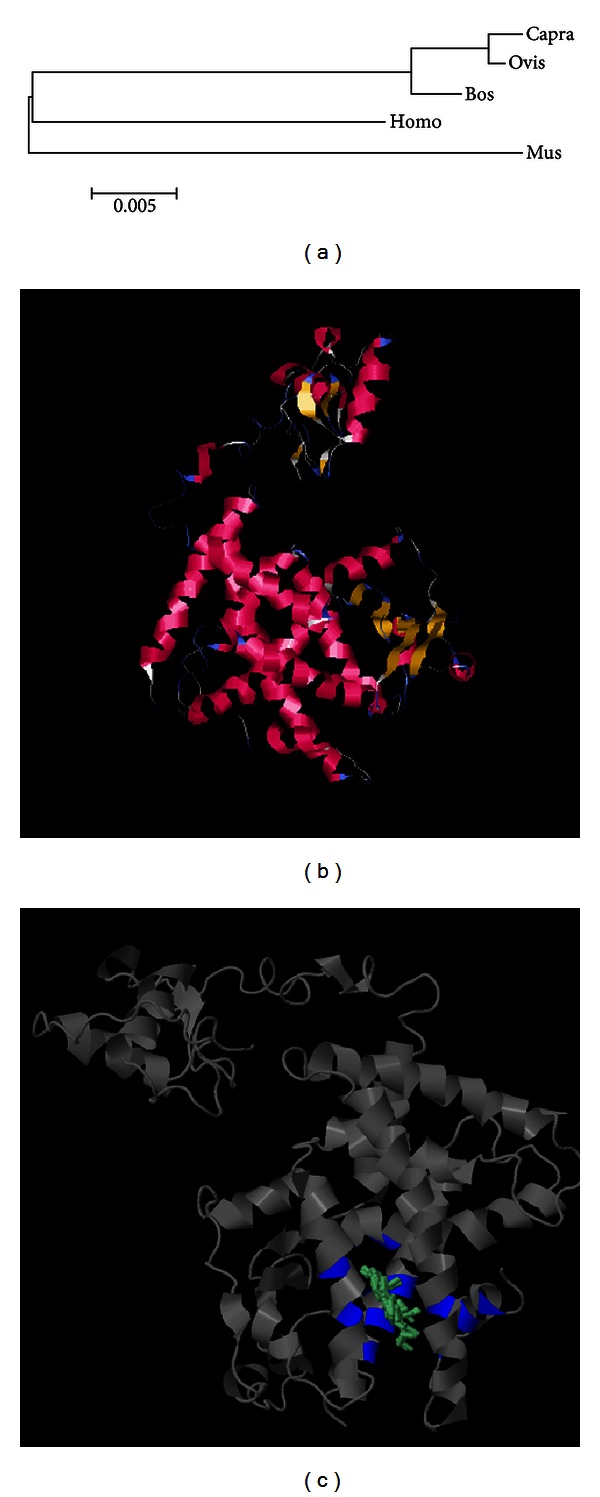
Structure prediction and phylogenetic alignment analysis of the dairy goat PPAR*γ* gene. (a) Phylogenetic tree showing the relatedness of PPAR*γ* CDS sequences of mouse (Mus), human (Homo), bovine (Bos), sheep (Ovis), and goat (Capra). The alignment was performed with ClustalW. The digital “0.005” is the genetic ruler. (b) The tertiary structure prediction of goat PPAR*γ*. Alpha helices are colored in crimson, beta sheets in yellow, turnings in blue, and irregular curl in white. (c) The ligand binding domain prediction of goat PPAR*γ*. The amino acids involved in the binding sites are colored in blue. The ligands colored in laurel green. In grey is the predicted tertiary structure of the goat PPAR*γ* protein.

PPAR*γ* is a member of the nuclear hormone receptor superfamily of transcription factors. It has been fully confirmed in humans and mice that PPAR*γ* directly regulates adipose cell proliferation, maturation, and differentiation [16, 17]. A potential role of PPAR*γ* in controlling milk fat synthesis also has been reported in bovine due to the increase of its expression between pregnancy and lactation [2] and the increase in expression of genes involved in milk fat synthesis after activation with ROSI [5]. However, its role, if any, on milk fat synthesis in the mammary gland of the goat remains relatively unknown. In this study, we cloned the dairy goat PPAR*γ* CDS and then used 5′RACE and 3′RACE procedures to obtain the full-length cDNA. The whole goat PPAR*γ* gene contains a 5′UTR of 114 bp, an ORF of 1428 bp, and a 3′UTR 215 bp. Homology alignment (BLASTN) revealed that the dairy goat PPAR*γ* gene (HQ589347.1) shares 90%, 89%, 98% and 98% identity with human (AB472042), mouse (NM_001127330.1), sheep (NM_001100921), and bovine (BC116098), respectively.  Figure 1(a) shows their genetic relationship. The structure prediction using online software revealed that there are two zinc finger structures and a ligand binding domain in the dairy goat PPAR*γ* protein (Figure 1(b)). It was also predicted (PredictNLS online software) that the nuclear localization signal sequence (*-*KKSRNKC*-*) of the dairy goat PPAR*γ* gene does not exist in either ends of the peptide chain, but it is present in the protein internal compartment. 

### 3.2. A PPAR*γ* Ligand Enhanced Activity of PPAR*γ* Response Element in GMECs

**Figure 2:**
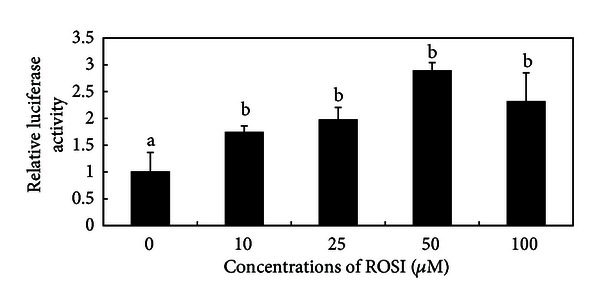
ROSI activated the PPAR*γ* response element (PPRE) effectively in GMECs. DMECs were transfected with pGL3-basic-PPRE×3 and pRL-TK vectors. After transfection, cells were treated with different concentration of ROSI. Luciferase and *Renilla* luciferase assays were performed in triplicate, and the results were expressed relative to the control (0 *μ*mol/L). Luciferase activity data were normalized with *Renilla* luciferase activity. The data represent mean ± SD of three independent experiments. ^b^ *P* < 0.05 versus the control group.

PPAR*γ* is a ligand-dependent nuclear transcription factor, and several unsaturated fatty acids in mammalian tissue are its natural ligands [18]. Binding of ligands to the PPAR*γ* ligand binding domain causes conformational changes in the receptor [16, 19]. Once activated, PPAR*γ* forms a heterodimeric complex with retinoid X receptor (RXR) and binds to the PPRE upstream of target genes [8]. In the present study, dairy goat mammary epithelial cells were incubated with rosiglitazone, a chemosynthetic ligand, which has a high affinity for PPAR*γ* and enhanced its activity. As shown in Figure 2, treatment with ROSI caused an activation of PPAR*γ* in GMECs. The luciferase levels between the treatment group and the control group (treatment with 0 *μ*mol/L ROSI) were statistically significant (*P* < 0.05). Data also indicated that the activation of the PPAR*γ* by ROSI reached a peak at 50 *μ*mol/L dose.

### 3.3. Activation of PPAR*γ* by ROSI Affects Expression of Genes Related to Triacylglycerol Synthesis and Lipid Droplets in GMECs

**Figure 3:**
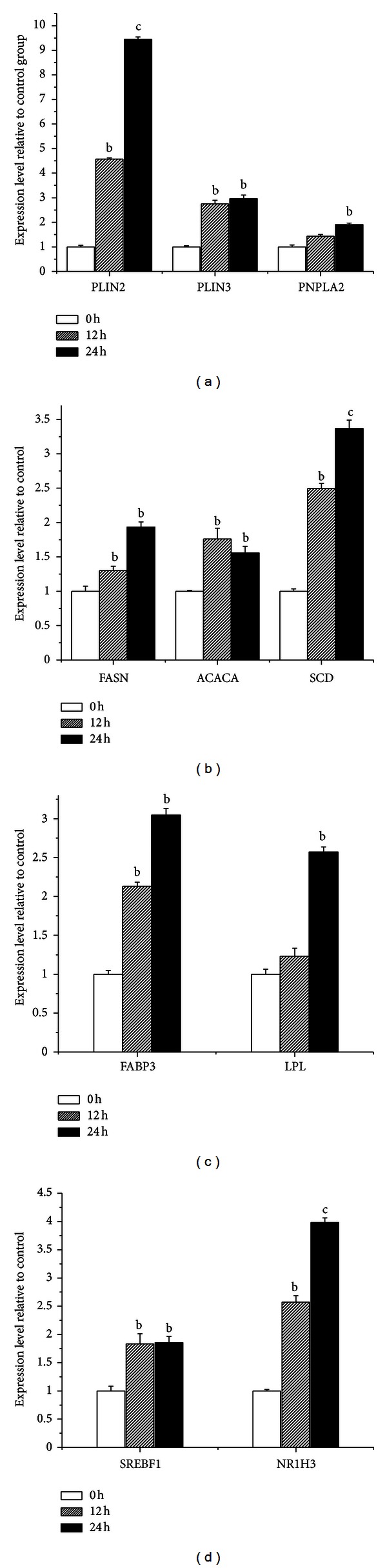
ROSI affects the expression of genes coding for proteins involved in lipid synthesis in GMECs through PPAR*γ* signaling. Dairy goat mammary epithelial cells were treated with ROSI and harvested at 0, 12, and 24 h. (a) Genes related to lipid droplet formation (*PLIN2* and *PLIN3*) and hydrolysis of triacylglycerols (*PNPLA2*). (b) Genes related to fatty acid synthesis (*FASN* and *ACACA*) and desaturation (*SCD*). (c) Genes related to cellular fatty acid uptake (*FABP3*, *LPL*). (d) Genes related to regulation of transcription (*SREBF1* and *NR1H3*). The data are mean ± SD of three independent experiments. ^b^ *P* < 0.05 versus the control group (0 h). ^c^ *P* < 0.01 versus the control group (0 h).

Genes related to *de novo* fatty acid synthesis (acetyl-coenzyme A carboxylase alpha (*ACACA*), fatty acid synthase (*FASN*)), desaturation (Stearoyl-CoA desaturase (*SCD*)), TAG synthesis (Diacylglycerol acyl transferase 1, (*DGAT1*)) and other genes including fatty acid binding protein 3 (*FABP3*) and Perilipin2 (*PLIN2*) were upregulated in adipose tissue of rats [20], humans [21], and bovine mammary epithelial cells [5] treated with ROSI. As summarized in Figure 3, treatment with ROSI increased the expression of *ACACA*, *FASN*, *SCD, FABP, LPL, *and also those associated with lipid droplet formation and hydrolysis (*PLIN2 *and patatin-like phospholipase domain containing 2, *PNPLA2*), and transcription regulators (*SREBF1*; liver X receptor *α*, *NR1H3*) (Figure 3). The significant (*P* < 0.05) increase in gene expression suggests that these genes are putative PPAR*γ* target genes in goat mammary gland. In a previous study, the expression of genes associated with long-chain fatty acid uptake or intracellular activation and transport, including *LPL*, was not affected by ROSI treatment of bovine mammary cells for 12 h [5]; however, our results revealed that *LPL *was upregulated significantly with ROSI treatment but only after 24 h. These contrasting responses may be related at least in part with inherent species differences in the regulatory mechanism via PPAR*γ* [5, 22].

### 3.4. Preliminary shRNA Screening and Adenovirus Generation

**Figure 4:**
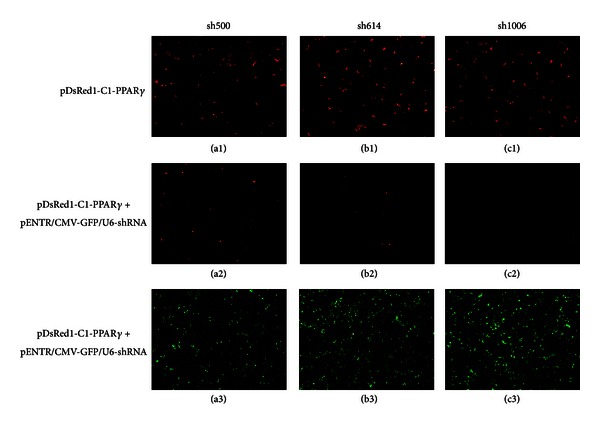
Efficacy screening of the three designed shRNA via images analysis. *pDsRed1-C1-PPAR*γ** vector was transfected as a control ((a1), (b1), and (c1)). The three tested shRNA (sh500, sh614, and sh1006) as *pENTR/CMV-GFP/U6*-shRNA construct were cotransfected with *pDsRed1-C1-PPAR*γ**vector. The transduction efficiency was estimated by the level of green fluorescent protein (GFP) expression ((a3), (b3) and (c3)). Shown are representative images of the PPAR*γ* expression (in red) after a 48 h cotransfection. (a1), (b1), and (c1) show high transfection and expression of PPAR*γ* construct vector. (a2), (b2), and (c2) show reduction of PPAR*γ* expression after addition of shRNA construct, while (a3), (b3), and (c3) show efficacy of shRNA transfection as shown by the green color (i.e., GFP). Images were obtained by a fluorescence microscope (Leica, DMI4000B, Germany) at 100x magnification. The images clearly show that the sh1006 had the highest effect on PPAR*γ* vector expression (c2).

The GFP protein on the *pENTR/CMV-GFP/U6-shRNA* vector was used to assess the efficacy of transduction via intensity of green fluorescence inside the cells. The 293A cells were either transfected with only the* pDsRed1-C1-PPAR*γ** construct (red fluorescent cells) or cotransfected with both constructs. Once the shRNA enters the cell, if specific for PPAR*γ*, it would enhance *pDsRed1-C1-PPAR*γ** construct with a concomitant reduction of red fluorescence. In this way, the shRNA efficacy in knocking down PPAR*γ* was assessed by the disappearance of red fluorescence in the cells. As shown in Figure 4, sh1006 and sh614 were more efficient than sh500 to silence PPAR*γ* (Figures 4(b2) and 4(c2)). There was more dsRED fusion protein being coded and detected in the sh500 group (Figure 4(a2)), indicating that sh500 had weaker silencing effect on goat *PPARG*. This was probably also due to the lower transfection observed for the sh500 construct (Figure 4(a3)). Although the approach depicted in Figure 4 is not quantitative, it represents a relatively easy way to screen efficient shRNAs.

**Figure 5:**
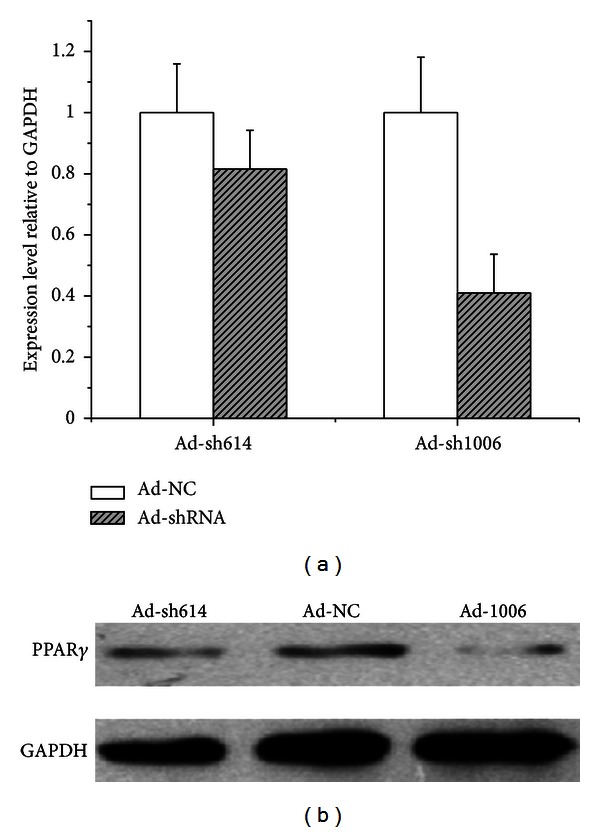
Efficacy screening of the two designed shRNA via RT-qPCR and western blot. The efficiency of Ad-sh614 and Ad-sh1006 (transduced with two adenoviruses at 200 multiplicity of infection for 48 h) in decreasing *PPARG* expression in dairy goat mammary epithelia cells was assessed by RT-qPCR (a) and western blot (b). The data revealed that Ad-sh1006 had the highest knockdown of PPAR*γ* transcript and protein; thus, it was used in the subsequent experiments.

According to the results of the preliminary screening, sh1006 and sh614 were selected to generate adenovirus Ad-sh614 and Ad-sh1006. Judging by the RT-qPCR and western blot analysis (Figure 5), compared with Ad-sh614 (about 20%), the Ad-sh1006 (about 60%) was more efficient in knocking down goat *PPARG*. 

### 3.5. Knockdown of Goat PPAR*γ* in GMECs Affects Expression of Genes Involved in Triacylglycerol Synthesis and Lipid Droplet Formation in GMECs

**Figure 6:**
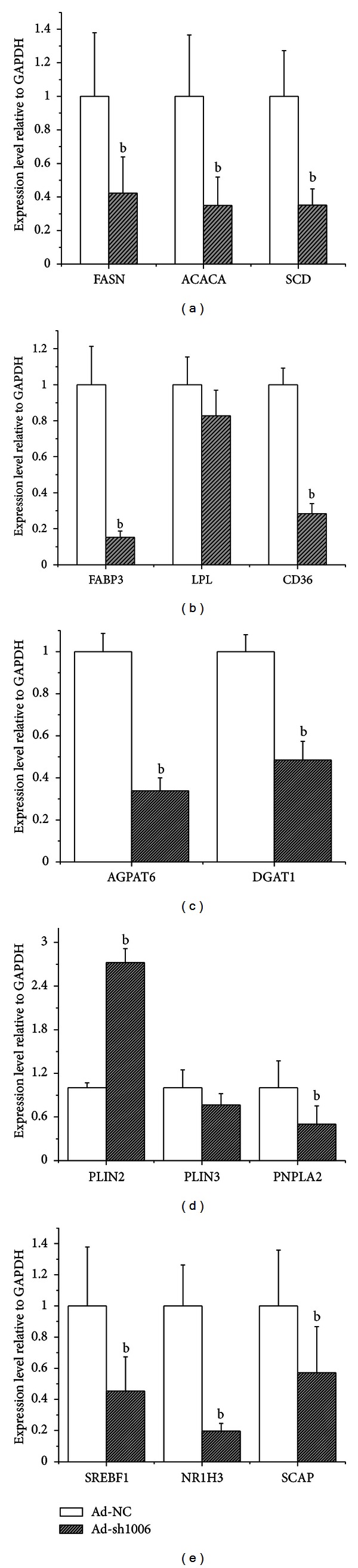
Effect of PPAR*γ* knockdown on genes coding for proteins involved in milk fat synthesis in GMECs. The expression of genes related to fatty acid synthesis (a), cellular fatty acid uptake (b), triacylglycerol synthesis (c), lipid droplet formation and triacylglycerol hydrolysis (d), and transcriptional regulation (e) was assessed in goat epithelial cells (GMECs) after transduction with Ad-sh1006 at 200 MOI for 48 h. The data represent the mean ± SD of cells transfected with control (Ad-NC) or Ad-sh1006 vector in triplicate per experiment. ^b^ *P* < 0.05 versus the control group.

Based on the above results, the Ad-sh1006 was selected to block expression of *PPARG* in GMECs, and expression analysis of genes known to be involved in milk fat synthesis and lipid droplet formation was evaluated (Figure 6). Results demonstrated that *FASN *(−58%), *ACACA *(−65%), and *SCD* (−65%) decreased significantly after *PPARG* knockdown (Figure 6(a)). With the exception of *SCD*, those data are in agreement with observations in bovine [5] and suggest that PPAR*γ* regulates *de novo *fatty acid synthesis and desaturation in goat mammary cells.

In bovine mammary cells, SREBF1 has attracted much attention because of its regulation of FASN and SCD expression and the major role played in milk fat synthesis [6, 23]. PPAR*γ* indirectly regulates SREBP1 protein activity through regulation of the expression of insulin-induced gene 1 (INSIG1) and directly regulates SREBF1 expression in adipose cells of mice [4]. We observed that the expression of *SREBF1* and *SCAP* decreased by 50% and 43% after knockdown of *PPARG* (Figure 6(e)). The mRNA of NR1H3 gene also was reduced by 75% when *PPARG* was knocked down (Figure 6(e)). Our data agree to a large extent with a previous bovine study, where an increase of *SREBF1* expression after ROSI treatment was observed [5]. We speculate that there might be two different signaling networks regulating *de novo* fatty acid synthesis in ruminant mammary cells. One pathway is under direct regulation of PPAR*γ* and encompasses genes such as *LPL, NR1H3*, and *FABP3 *(Figures 6(b) and 6(e)); another is under indirect regulation of PPAR*γ* through SREBF1 and NR1H3 (Figure 6(e)) which would, in turn, participate in upregulation of the transcription of *FASN* and *ACACA *[23–25]. Regardless of the specific mechanism, our data support the previous hypothetical milk fat synthesis transcriptional networks proposed for bovine mammary [3]. In agreement with that previous proposal, our data support a complex regulatory network that controls mammary triacylglycerol synthesis in goat mammary cells such that several protein factors serve as putative checkpoints to regulate milk fat synthesis. PPAR*γ* appears to be one of those factors in dairy goats.

PPAR*γ* plays multifaceted roles in the regulation of triacylglycerol synthesis and secretion besides the *de novo* synthesis of fatty acids. As an adiposity factor, PPAR*γ* is able to regulate triacylglycerol synthesis and deposition and then dominate the process of differentiation of fat cells [26]. In the present study, the mRNA expression of genes related to triacylglycerol synthesis *DGAT1* (−52%) and *AGPAT6 *(−67%) decreased greatly after infection with Ad-sh1006 (Figure 6(b)), which suggests that PPAR*γ* regulates triacylglycerol synthesis in mammary cells as in fat cells.

Triacylglycerols are deposited in fat cells, while in the mammary cells they are secreted in the form of lipid droplets in milk. To investigate the role of PPAR*γ* in transcription of milk fat globule protein genes, we measured the mRNA expression of* PLIN2, PLIN3, *and* PNPLA2 *after PPAR*γ* knockdown (Figure 6(d)). The expression of *PLIN2* was largely induced while the expression of *PLIN3 *and *PNPLA2 *decreased approximately 24% and 50%, respectively, in cells transfected with Ad-sh1006, while it is not extremely for *PLIN3*. Previous data from humans [27] indicated that there is a PPRE on the promoter of the PLIN2 gene; thus, it is considered as a downstream target and would be decreased after PPAR*γ* knockdown. However, our data showed that the expression of* PLIN2 *had an unexpected increase. Such response might have been caused by compensatory effects of other unidentified transcription factors.

Other data also support the evidence [28] that PPAR*γ* could affect not only the genes related to fatty acid transport, but also genes that control triacylglycerol hydrolysis in goat mammary cells (Figure 6(c)). For instance, expression of *PNPLA2* is significantly increased during lactation in bovine mammary tissue [29]. However, judging by differences in milk fatty acid profiles between goat milk and bovine milk [30], goat mammary lipid synthesis differs in some respects from bovine. From a mechanistic standpoints the upregulation of *PNPLA2* after PPAR*γ* activation may be functionally related with the unique characteristics of goat milk.

Our data showed that even if there is great similarity between two ruminant dairy species such as goat and cow [5], there are still some inherent differences between them. Such differences may at least in part be caused by different target genes of PPAR*γ* in each species. Attempts to compare *in vitro* data among studies performed in different laboratories are obviously challenging because of differences in cell culture conditions (e.g., culture medium, absence of prolactin in our study and not in bovine [31]) and also different protocols. The comparisons of data from the present study with data generated in bovine mammary [31] are likely also slanted because of the use in the present study of *GAPDH* as the only internal control for RT-qPCR normalization versus multiple genes used in the bovine study.

## 4. Conclusions

In the present studies, we cloned the PPAR*γ* gene in dairy goat mammary gland and explored its function *in vitro*. As proposed in bovine mammary gland, PPAR*γ* plays a multifaceted role in regulating the overall process of fatty acid and triacylglycerol synthesis and secretion. Our overall data indicate that PPAR*γ* in goat mammary plays a role in controlling milk fat synthesis directly or via the activation of the transcription regulators *SREBF1* and *NR1H3*. Together, our data provide strong evidence that PPAR*γ* is the key regulator of milk fat synthesis in ruminants. Hence, controlling PPAR*γ* activation may prove useful in regulating milk fat production in the lactating dairy goat.

## Figures and Tables

**Table 1:** Primer pairs used in PCR for amplification of goat *PPARG* from mammary cDNA.

Name of fragment	Sequence	Product length
PPAR*γ* CDS	Forward: 5′-ATGGTTGACACAGAGATGCCG-3′	1413 bp
	Reversal: 5′-GTAGATTTCCTGTAGAAGTGGGTGG-3′	
PPAR*γ* 3′RACE	Outer: 5′-AAGTAACTCTCCTAAAATACGGCG-3′	516 bp
	Inner: 5′-CCAGAAAATGACGGACCTCAGGCAGA-3′	160 bp
PPAR*γ* 5′RACE	GSP1: 5′-CGGTGATTTGTCTGTCGTCTTTC-3′	750 bp
	GSP2: 5′-GATACAGGCTCCACTTTGATTGC-3′	260 bp

**Table 2:** Characteristics of shRNA used in the experiment.

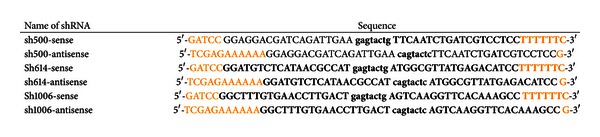

Three shRNAs (numbers stand for their position in cDNA) were designed, and each shRNA was added with restriction sites *BamH* I and *Xho* I. The loop domain (lower-case nucleotides) contained a *Scal* I site.

**Table 3:** Characteristics of primer pairs used, amplicon length, and efficiency of reaction in the RT-qPCR.

Accession^#^	Gene	Primer sequence (5′ to 3′)	Product length (bp)	Efficiency
JN236219.1	*ACACA *	Forward: CTCCAACCTCAACCACTACGG Reversal: GGGGAATCACAGAAGCAGCC	171	2.09
JI861797.1	*AGPAT6 *	Forward: AAGCAAGTTGCCCATCCTCA Reversal: AAACTGTGGCTCCAATTTCGA	101	2.17
X91503^#^	*CD36 *	Forward: GTACAGATGCAGCCTCATTTCC Reversal: TGGACCTGCAAATATCAGAGGA	81	2.18
DQ380249.1	*DGAT1 *	Forward: CCACTGGGACCTGAGGTGTC Reversal: GCATCACCACACACCAATTCA	101	2.11
NM_001009350	*FABP3 *	Forward: GATGAGACCACGGCAGATG Reversal: GTCAACTATTTCCCGCACAAG	120	2.14
DQ915966.3	*FASN *	Forward: GGGCTCCACCACCGTGTTCCA Reversal: GCTCTGCTGGGCCTGCAGCTG	226	2.13
AJ431207	*GAPDH *	Forward: GCAAGTTCCACGGCACAG Reversal: GGTTCACGCCCATCACAA	249	2.16
DQ997818	*LPL *	Forward: AGGACACTTGCCACCTCATTC Reversal: TTGGAGTCTGGTTCCCTCTTGTA	169	2.18
GU332719	*NR1H3 *	Forward: CATCAACCCCATCTTCGAGTT Reversal: CAGGGCCTCCACATATGTGT	163	2.13
HQ846826	*PLIN2 *	Forward: TACGATGATACAGATGAATCCCAC Reversal: CAGCATTGCGAAGCACAGAGT	203	2.16
HQ846827	*PLIN3 *	Forward: GGTGGAGGGTCAGGAGAAA Reversal: TCACGGAACATGGCGAGT	170	1.13
GQ918145	*PNPLA2 *	Forward: GGAGCTTATCCAGGCCAATG Reversal: TGCGGGCAGATGTCACTCT	226	2.24
HQ589347.1	*PPARG *	Forward: CCTTCACCACCGTTGACTTCT Reversal: GATACAGGCTCCACTTTGATTGC	145	2.21
DV935188^#^	*SCAP *	Forward: CCATGTGCACTTCAAGGAGGA Reversal: TGTCGATCTTGCGTGTGGAG	108	2.10
GU947654	*SCD *	Forward: CCATCGCCTGTGGAGTCAC Reversal: GTCGGATAAATCTAGCGTAGCA	257	2.10
HM443643.1	*SREBF1 *	Forward: CTGCTGACCGACATAGAAGACAT Reversal: GTAGGGCGGGTCAAACAGG	81	2.20

Annealing temperature for all primers in this table is 60°C.

*ACACA*, acetyl-coenzyme A carboxylase alpha; *AGPAT6*, 1-acylglycerol-3-phosphate O-acyltransferase 6; *CD36*, thrombospondin receptor; *DGAT1*, diacylglycerol acyl transferase 1; *FABP3, *fatty acid binding protein 3; *FASN*, fatty acid synthase; *GAPDH*, glyceraldehyde-3-phosphate dehydrogenase; *LPL*, Lipoprotein lipase; *NR1H3*, liver X receptor *α*; *PLIN2*, perilipin2; *PLIN3*, perilipin3; *PNPLA2*, patatin-like phospholipase domain containing 2; *PPARG*, peroxisome proliferator-activated receptor *γ; SCAP*, cleavage activating protein; *SCD*, stearoyl-CoA desaturase; *SREBF1*, Sterol regulatory element-binding transcription factor 1.

^#^The primer sequences are from bovine.
